# Protocol for EHS Guideline on Treatment of Ventral and Incisional Hernias in Emergency

**DOI:** 10.3389/jaws.2025.14644

**Published:** 2025-10-01

**Authors:** Sara Capoccia Giovannini, Maciej Pawlak, Stavros A. Antoniou, Heather Bougard, Umberto Bracale, Eva Deerenberg, René H. Fortelny, Christine Gaarder, Miguel Ángel García-Ureña, Katie Gilmore, Sergio Alejandro Gomez-Ochoa, Ferdinand Köckerling, Ana Pilar Morante Perea, Yohann Renard, José A. Pereira Rodriguez, Benoît Romain, Andrea Carolina Quiroga-Centeno, Sebastian Schaaf, Elena Schembari, Alexis Theodorou, Cesare Stabilini, Francesca Pecchini

**Affiliations:** ^1^ Department of Surgery, Policlinico San Martino IRCCSUniversity of Genoa, Genoa, Italy; ^2^ Department of Surgical Sciences, University of Genoa, Genoa, Italy; ^3^ Department of General & Abdominal Wall Surgery, Golden Jubilee National University Hospital, Glasgow, United Kingdom; ^4^ School of Medicine, Dentistry and Nursing, University of Glasgow, Glasgow, United Kingdom; ^5^ Medical School, European University Cyprus, Nicosia, Cyprus; ^6^ Department of Surgery, New Somerset Hospital and University of Cape Town, Cape Town, South Africa; ^7^ Department of Gastroenterology, Endocrinology and Endoscopic Surgery, University Hospital of Naples, Federico II, Naples, Italy; ^8^ Department of Surgery, Franciscus Gasthuis en Vlietland, Rotterdam, Netherlands; ^9^ Medical Faculty, Sigmund Freud Private University Vienna, Vienna, Austria; ^10^ Institute of Clinical Medicine, University of Oslo, Oslo, Norway; ^11^ Department of Traumatology, Oslo University Hospital, University of Oslo, Oslo, Norway; ^12^ Grupo de Investigación de Pared Abdominal Compleja, Facultad de Medicina, Universidad Francisco de Vitoria, Hospital Universitario del Henares, Madrid, Spain; ^13^ Heart Failure and Transplant Clinic, Fundación Cardiovascular de Colombia, Floridablanca, Colombia; ^14^ Department of General Internal Medicine and Psychosomatics, Heidelberg University Hospital, Heidelberg, Germany; ^15^ Hernia Center, Vivantes Humboldt-Hospital, Academic Teaching Hospital of Charité University Medicine, Berlin, Germany; ^16^ Hospital Universitario Fundación Alcorcón, Madrid, Spain; ^17^ Reims Champagne-Ardennes, Department of General, Digestive and Endocrine Surgery, Robert Debré, University Hospital, Reims, France; ^18^ Abdominal Wall Surgery Unit, Section of General Surgery, Department of General Surgery, Parc de Salut Mar, Hospital del Mar Medical Research Institute (IMIM), Barcelona, Spain; ^19^ Department of Digestive Surgery, Centre Hospitalier Universitaire de Strasbourg, Strasbourg, France; ^20^ Department of Surgery, Universidad Industrial de Santander, Bucaramanga, Colombia; ^21^ School of Translational Medicine, Medical Faculty Mannheim, Heidelberg University, Mannheim, Germany; ^22^ Department of General, Visceral and Thoracic Surgery, German Armed Forces Central Hospital Koblenz, Koblenz, Germany; ^23^ Department of Colorectal Surgery, Royal Devon and Exeter Hospital, Exeter, United Kingdom; ^24^ 1st Department of Surgery, Hippocratio Hospital, University of Athens, Athens, Greece; ^25^ Department of General Surgery, Emergency and New Technologies, Baggiovara General Hospital, AOU Modena, Modena, Italy

**Keywords:** protocol, guidelines, incisional hernia, ventral hernia, emergency hernia surgery

## Abstract

**Background:**

Emergency presentations of ventral and incisional hernias are common and associated with significantly higher morbidity and mortality than elective procedures. Despite their frequency, clinical guidance on managing acutely complicated hernias in emergency settings remains limited and heterogeneous.

**Objective:**

This protocol outlines the development of an evidence-based clinical guideline by the European Hernia Society (EHS) to support general and emergency surgeons in treating acutely complicated ventral and incisional hernias in adults.

**Methods:**

Developed according to AGREE-S and GRADE methodologies, this guideline will address seven key clinical questions concerning the use of mesh, surgical approach, and technique based on clinical contamination status and anatomical considerations. A steering group of hernia surgery experts and methodologists leads the project, supported by two independent evidence review teams. Patient input will be obtained via structured surveys to incorporate values and preferences. Systematic literature reviews, evidence-to-decision frameworks, and consensus meetings will guide the formulation of recommendations, with strength and direction defined according to GRADE standards.

**Discussion:**

This is the first EHS guideline specifically targeting the emergency management of incisional and ventral hernias. By addressing urgent clinical decisions through a structured, transparent, and inclusive process, it aims to reduce variability in care, improve outcomes, and serve as a resource for clinical practice and future research. The guideline will be published open access and monitored for implementation and updates based on emerging evidence.

## Introduction

Abdominal wall hernia is one of the most common conditions encountered by general surgeons [1].

Rising rates of abdominal wall hernia repair have been described in current literature however, population-based evidence concerning incidence rates of emergent hernia repair and changes with time are not completely known [2].

Acutely symptomatic abdominal wall and groin hernias (ASH) are a common reason for emergency surgical admission and emergency presentations account for around 25 per cent of all hernia repairs [3].

Outcomes of emergent ventral and incisional hernia repair, in consideration to the poor clinical status of the patient and the possible presence of contamination, are worse than elective, a ten-fold increase in mortality has been reported as well as increased morbidity rates [4] as expected for a complex abdominal wall defect repair [5].

Accordingly, the emergency treatment of ventral and incisional hernia poses specific problems in comparison to elective repair due to the complete difference in approach strategies and the priority of expected results. While the elective treatment aims to improve the patient’s quality of life, prevent acute complications, and includes preoperative optimisation of comorbidities [6, 7], the emergency treatment, conditioned by time, aims to safeguard the life of patients who cannot be prehabilitated, preserves the integrity of the digestive system, and, only finally, reconstructs the abdominal wall.

Emergency presentations frequently expose surgeons to a spectrum of clinical scenarios within a single pathology, necessitating management distinct from elective interventions. Treatment variations, often influenced by timing, patient status, and presenting emergency, contribute to considerable heterogeneity across practices and centers [8], *potentially yielding inconsistent surgical outcomes*. This underscores the need for standardized therapeutic responses and treatment protocols to optimize patient safety and care, especially given the unequivocal priority of life preservation in emergency surgical contexts.

The European Hernia Society (EHS) developed the ENGINE project to establish a rigorous pathway for guideline production and updating, consistently following the GRADE methodology beneficial for patients and surgeons [9, 10].

This current guideline is the first to be conceived and conducted entirely according to this structured project. Within this comprehensive methodology, the initial step involves conducting a scoping review, a particular type of systematic review crucial for mapping the current landscape of literature on a specific topic and identifying gaps in primary research [11]. This is followed by the formulation of relevant PICO (Population, Intervention, Comparison, Outcome) Key Questions (KQs), the conduction of meta-analyses for each KQ, and Evidence to Decision (EtD) evaluations. These steps culminate in the formulation of evidence-based recommendations, which are then proposed to panel members for voting to achieve consensus prior to dissemination, as was recently done for the management of Diastasis Recti in Post-gravidic Women [12].

## Main and Secondary Hypothesis

In light of the current developments in terms of technique and materials available for abdominal wall reconstruction, the objective of this guideline is to provide transparently developed, trustworthy, and evidence-informed recommendations on the treatment of acutely complicated ventral and incisional hernias in adult patients, in particular helping general and emergency surgeon to navigate the main treatment choice of this difficult clinical scenario.

Importantly, this guideline is designed to complement existing EHS guidelines, particularly those on midline incisional hernia repair [13] and management of the abdominal wall in the context of the open or burst abdomen [14], by focusing specifically on emergency presentations, which were only briefly addressed in those prior documents.

## Methods and Design

The present protocol conforms to AGREE-S and PRISMA-P reporting standards [15, 16]. It will be made available on the EHS website for healthcare professionals to access, and EHS members will be invited through various channels (social media, email newsletter) to comment on the content. After exploring literature status through a Scoping Review on Emergency ventral and inguinal hernia management [17], a series of 7 key questions have been developed, discussed and agreed by the guideline development group in a meeting held during the annual Congress of EHS in Prague 2024. Additionally, an online survey among EHS members will be conducted on the key questions (KQ) chosen by the steering group (SG) to inform the panel of the current practices in the emergent treatment of abdominal wall defects. Relevant comments will be considered. Patients’ involvement will be sought by means of an online survey to ensure that their preferences were considered in the decision-making process and incorporated into the Guideline development.

### Steering Group

The steering group will consist of 4 general and abdominal wall surgeons with specific interest in hernia surgery, former members of the EHS Board, and experts in guideline development and evidence synthesis. They are free of COI as per GRADE recommendation.

### Guideline Methodologist

One of the members of the steering committee will be an INGUIDE-certified methodologist (INGUIDE certificate number 2021-L2-V1-00001).

### Evidence Review Team

Two evidence review teams (ERT) are created, one dedicated to the systematic search of literature of interventional trials from which evidence on available treatment can be extracted. The second team is dedicated to finding evidence informative of the items included in the Evidence to Decision Framework (Burden and relevance of the condition, benefit and harms of the treatments, resource use, acceptability, equity, patients’ values, feasibility, and cost-effectiveness of the treatments). Each evidence outreach team will consist of two healthcare professionals with experience in evidence outreach, senior and junior, to maximise the experience gained. They will be free of direct and indirect conflicts, and they will act independently from the steering group, they will, however, consult the guideline methodologist and the guideline panel, as recommended in GRADE standards.

### Guideline Panel and External Advisors

The guideline panel will consist of four general surgeons, one emergency/trauma surgeon, three general surgeons with a specific interest in hernia surgery, one anaesthetist and two patient representatives. The composition of the panel aims to ensure representation of both genders, international diversity, and both academic and non-academic practice. Every member of the panel will receive training and certification through the INGUIDE program (level 1 certification–panel member).

Input will be asked from surgeons who have published RCTs, meta-analyses and/or published guidelines on the topic of emergent treatment of ventral and incisional hernia. However, they will not have voting privileges on the direction and the strength of the recommendations within the evidence-to-decision framework due to indirect conflicts, as per Guidelines International Network guidelines [18]. The guideline panel’s and external advisors’ contributions will be acknowledged by authorship in the resulting journal publication.

### PICO Questions

A series of 7 key questions have been developed, discussed and agreed upon by the guideline development group in a meeting held during the annual Congress of EHS in Prague 2024. Additionally, an online survey among EHS members will be conducted on the key questions (KQ) chosen by the steering group (SG) to inform the panel of the current practices in the emergent treatment of abdominal wall defects. Relevant comments will be considered.

KQ 1 – should mesh vs. tissue repair be used for the emergency treatment of ventral and incisional hernia in CDC 1, stable patients with a defect amenable to direct closure?

KQ 2 – should mesh vs. tissue repair be used for the emergency treatment of ventral and incisional hernia in CDC ≥ 2, stable patients with a defect amenable to direct closure?

KQ 3 – should mesh vs. tissue repair be used for the emergency treatment of ventral and incisional hernia in CDC 1, stable patients with a defect not amenable to direct closure?

KQ 4 – should mesh vs. tissue repair be used for the emergency treatment of ventral and incisional hernia in CDC ≥ 2, stable patients with a defect not amenable to direct closure?

KQ 5 – should sublay mesh placement vs. other position mesh placement be used for the emergency repair of acutely complicated ventral and incisional hernia.

KQ 6 – Should synthetic mesh vs. other mesh type be used for emergency treatment of adult patients with acutely complicated ventral and incisional hernia.

KQ 7 – Should laparoscopic approach vs. open be used for emergency treatment of adult patients with acutely symptomatic/complicated ventral and incisional hernia in a stable patient.

### Guideline Development Methodology

The guideline project will follow the principles of the AGREE-S checklist [15] and GRADE guideline development methodology [19] and will adhere to the process as summarised by the Guidelines International Network [20]. The guideline panel will be asked to comment on the Key questions and planned analyses. The steering group will draft a list of potential outcomes. To identify outcomes of interest, panel members will independently assess the importance of each outcome using the GRADE scale. They will also have the opportunity to propose additional outcomes considered significant or critical. The steering group will calculate the median score for each outcome to identify important (score 4–6) and critical (score 7–9) items for inclusion. If substantial variation is obtained among panel evaluations of outcomes, a synchronous meeting will be held online to resolve conflicts and reach a consensus. A pre-defined consensus threshold of ≥70% agreement will be used to approve recommendations during panel meetings and Delphi rounds. This discussion will be guided by placing emphasis on patient-important outcomes while adhering to Cochrane guidance in focusing on the most relevant outcomes for patients, clinicians, and policymakers.

The literature search strategy will be developed by two members of the steering committee together with the librarian and with the help of the senior member of the ERT with experience in outreach, knowledge, and evidence search, and it will be built *de novo*.

A comprehensive search from 2000, through the healthcare database will include Pubmed and Scopus. The grey literature analysis will not be possible due to the unavailability of the OpenGrey repository. Relevant terms will be selected to identify eligible reports. Mesh terms, search operators, and search limits in each of the above databases will be adapted accordingly.

The risk of bias in eligible studies will be assessed per single outcome using RoB 2 for randomised trials [21]. In the case of non-randomised comparative studies, the last version of the ROBINS-I instrument will be adopted [22], and the JBI tool for bias assessment in case series will be used for single-arm papers [23].

Study selection will be performed by the evidence outreach team, and the subsequent risk of bias assessment and data extraction will be performed by the same team and independently cross-checked by the guideline methodologist.

Comparative studies published from January 1st, 2000, to November 30th, 2024, will be included if they contain extractable data on adult patients undergoing ventral hernia repair under emergency conditions, according to the specific features explored in the Key Questions (KQs). Studies reporting data on inguinal, groin, or parastomal hernia, open abdomen or burst abdomen closure, traumatic incisional hernia, and all patients treated under elective conditions will be excluded. While acknowledging the growing role of robotic platforms in abdominal wall repair [24] and their potential application in emergency settings, the Steering Group decided to exclude their use from the scope of these guidelines due to limited data availability and concerns regarding equity on their access for the patients.

Biostatisticians will perform statistical analyses using the methodology reported below.

GRADE evidence tables will be developed with GRADEpro GDT by the methodologists and revised by the panel during a face-to-face consensus meeting of the guideline development group. Draft recommendations will be formulated, and their strength will be defined. The recommendations may be refined, and their strength may be revised in line with the results of an online Delphi process of panel members. Comments by the Delphi panel must be in accordance with the GRADE methodology in order to be considered. The formulation of recommendations will be formulated as suggested by GRADE methodology [19].

The project will be managed using project managing software (ClickUp software–San Diego, CAL, USA).

### Data Collection and Management

Data will be extracted from eligible studies by the Evidence Review Teams (ERTs) using a standardised data extraction form. Extracted data will include study characteristics (e.g., design, sample size, intervention details, comparator details, outcome definitions, follow-up duration), participant characteristics (e.g., age, sex, comorbidities, hernia characteristics), and outcome data. The data will be entered into a secure, password-protected database, and double data entry will be performed to ensure accuracy. Any discrepancies will be resolved by consensus within the ERTs and overseen by the guideline methodologist.

### Effect Measures

The choice of effect measure for each outcome will be guided by the nature of the data and consistency of reporting across included studies. For binary outcomes (e.g., mortality, surgical site infection), relative risk (RR), odds ratio (OR), and risk difference (RD) will be considered. RRs and ORs are often preferred for their generalisability across different baseline risks, while RDs provide a more direct measure of absolute effect. If enough information is provided, RRs will be used as the default for all pooled analyses.

For time-to-event outcomes (e.g., hernia recurrence), hazard ratios (HRs) will be the preferred metric, as they account for the timing of events. For studies that report their findings through log-rank statistics, we will employ Parmar’s method to estimate hazard ratios. This approach utilises the observed minus expected (O-E) statistic and its variance (V) to compute the natural logarithm of the hazard ratio as (O-E)/V, with the standard error calculated as the square root of the reciprocal variance. When studies present their results through Kaplan-Meier curves rather than direct statistics, we will implement a systematic digitisation process (WebPlotDigitizer software, version 5), followed by the application of Guyot’s algorithm for individual patient data reconstruction. This reconstructed data will undergo validation through comparative analysis of the reported versus reconstructed survival estimates before being utilised in our meta-analytic procedures. If some studies provide the hazard ratio but others give risk ratios (or the number of events and sample size in each group), we will combine them in a sensitivity analysis.

For continuous outcomes (e.g., length of hospital stay, pain scores), mean difference (MD) will be used when outcomes are measured on the same scale across studies. If different scales are used, standardised mean differences (SMDs) will be employed to facilitate comparison. SMDs express the effect size in terms of standard deviations, allowing for the pooling of results from studies using different measurement instruments. For all outcomes, minimum clinically important differences (MCIDs) will be established *a priori* by the guideline panel to aid in interpreting the clinical significance of observed effects.

### Meta-Analysis

When two or more studies report data on the same outcome, a pairwise meta-analysis will be performed. Given the anticipated clinical and methodological diversity among studies, a random-effects model will be employed as the primary analysis approach. Specifically, both the restricted maximum likelihood estimator and the Paul-Mandel estimators will be used, and the results derived from each method will be compared to obtain a robust approach in the presence of heterogeneity. The random-effects model assumes that the true effect size varies between studies and estimates the average effect size and its uncertainty, acknowledging this between-study variability. For comparison and to assess the robustness of the findings, sensitivity analyses will be conducted using a fixed-effect model (Mantel-Haenszel method for dichotomous outcomes and inverse-variance method for continuous outcomes). The fixed-effect model assumes that all studies are estimating the same underlying true effect and that any observed differences are solely due to chance.

### Assessment of Heterogeneity

Heterogeneity between study results will be assessed using a combination of statistical tests and visual inspection. The I^2^ statistic will quantify the percentage of variation across studies attributable to heterogeneity rather than chance. Tau^2^ (between-study variance) will provide an estimate of the variability in true effect sizes between studies. Forest plots will visually represent the effect estimates and confidence intervals of individual studies, allowing for a graphical assessment of heterogeneity. Prediction intervals for each contrast will also be computed. If substantial heterogeneity (I^2^ ≥ 50%) is identified, potential sources will be investigated through pre-planned subgroup analyses and if sufficient studies are available (≥10), meta-regression analysis to investigate the relationship between the effect size and continuous or ordinal covariates (e.g., patient age, defect size), providing a more nuanced understanding of heterogeneity. On the other hand, subgroup analyses will involve stratifying the meta-analysis results based on categorical covariates (e.g., CDC classification, type of mesh). Statistical tests for subgroup differences (e.g., Cochran’s Q test) will be used to determine if the effect size differs significantly between subgroups.

### Handling of Missing Data

Missing data can introduce bias and reduce the precision of effect estimates. Therefore, every effort will be made to obtain missing outcome data by contacting study authors. If data remains unavailable, the potential impact of missing data will be thoroughly discussed and addressed through sensitivity analyses. For missing dichotomous outcome data, sensitivity analyses will be performed based on the approach described by Higgins et al. [25] which involves exploring different assumptions about the risk of the outcome in the missing participants relative to the observed participants (e.g., assuming all missing participants experienced the event, assuming none of the missing participants experienced the event). For continuous outcomes, imputation methods, such as multiple imputation, may be considered if appropriate. The robustness of the primary meta-analysis results to the chosen imputation model will be assessed through sensitivity analyses.

### Assessment of Publication Bias

In order to assess publication bias, funnel plots will be visually inspected when at least ten studies are available per outcome. A funnel plot graphs the effect size of each study against its precision (usually the standard error), with smaller studies expected to scatter more widely at the bottom of the plot. Asymmetry in the funnel plot can suggest publication bias, with studies showing no effect or effects opposite to the overall effect being underrepresented. If asymmetry is observed, statistical tests like Egger’s test will be performed to formally assess publication bias. If publication bias is suspected, its potential impact on the pooled effect estimate will be further explored using techniques like the trim-and-fill method, which imputes “missing” studies based on the observed asymmetry and recalculates the pooled effect.

### Network Meta-Analysis

When multiple interventions are being compared (e.g., different mesh types, different surgical approaches), network meta-analysis (NMA) offers a powerful tool to synthesise evidence from direct and indirect comparisons. If sufficient data are available, NMA will be considered to evaluate the relative effectiveness of all available interventions (e.g., mesh positioning) for emergency ventral and incisional hernia repair. NMA allows for the simultaneous comparison of all interventions within a single analysis, potentially providing more precise estimates of treatment effects and informing more comprehensive recommendations. The consistency of the network (i.e., the agreement between direct and indirect evidence) will be rigorously evaluated using established methods such as node-splitting. This involves splitting a node in the network (representing a particular comparison) and comparing the direct evidence for that comparison with the indirect evidence derived from other comparisons in the network.

### Statistical Software

Statistical analyses will be performed using R (version 4.4.2). Specifically, the following packages will be employed: meta (for standard pairwise meta-analysis), metaphor (for meta-regression and more advanced meta-analytic techniques), and net meta (for network meta-analysis). The specific functions and commands used for each analysis will be documented in the guideline.

### Target Users

This guideline is intended to be used by general and emergency surgeons and patients. The guideline publication will contain a short abstract in plain language for patients to use.

### Publication and Dissemination Strategy

As an EHS-sponsored project, this guideline will be submitted for publication in the Journal of Abdominal Wall Surgery, the official journal of the EHS.

### Feedback

The steering group will consider constructive feedback received during the project via various routes and sources, such as letters to the editor and social media. Such feedback will be taken into account in the guideline development process or in future updates.

### Monitoring, Update and Future Steps

Use of the guideline by EHS members will be monitored through an online survey 2 years after publication. The timing of the update of the guideline will be based on new research data on this topic.

## Discussion

### Implications for Practice and Research

Stringent criteria defined by GRADE and AGREE-S will be applied to collate, appraise and analyse the available evidence.

The proposed guideline is expected to inform decision-making and guide clinical practice and health policy. Guidance will be provided on direction and implications for future research in light of identified evidence gaps through a scoping review part of the present project.

This Clinical Guideline will address the treatment algorithm of an acutely complicated (acutely irreducible/strangulated) ventral and incisional hernia in adult patients requiring emergent treatment. It is expected to provide useful, evidence-based and stakeholder-informed information on the most appropriate management in this difficult clinical scenario.

### Strengths and Limitations

The proposed guideline has several strengths. First, the GDG will represent all groups of stakeholders involved in the diagnosis, management, and treatment of acutely complicated ventral and incisional hernias. Second, the authors will have the opportunity to survey a dedicated patient advisory group to explore patients’ values and preferences, and two patient representatives will be on the panel with the right to vote on recommendations. Third, the authors will use the GRADE approach to assess the certainty of evidence and formulate recommendations in a systematic and transparent manner.

A potential limitation is the lack of, or very scarce, evidence to develop clinical expert guidance and answer some of the key questions.

### Research Ethics

This project does not involve any intervention, identifiable human data, or animal subjects. Therefore, ethics approval and informed consent were not required, in accordance with institutional and journal guidelines. This is consistent with international standards for methodological research (AGREE-S, GRADE, and GIN) that do not require formal ethical approval for guideline protocols involving systematic review and expert consensus only.

EHS, as the funder, will not be involved in the development of this guideline despite the presence of two former members of the EHS Executive Board. Research Ethics Committee approval is not necessary as this project does not involve any identifiable patient data. Declaration of interest statements will be collected by all guideline panel members before and upon completion of the project. Panel members with direct conflicts will be replaced, and participants with indirect conflicts will be reassigned to functions according to the recommendations of the Guidelines International Network [26].

## Conclusion

This guideline, prepared in compliance with contemporary methodological standards and involving a multidisciplinary panel of experts, is designed to offer a therapeutic algorithm to assist general surgeons in making challenging decisions regarding the emergency management of abdominal wall defects.

## Data Availability

The original contributions presented in the study are included in the article/supplementary material, further inquiries can be directed to the corresponding author.
